# WU and KI Polyomaviruses in Respiratory Samples from Allogeneic Hematopoietic Cell Transplant Recipients

**DOI:** 10.3201/eid1810.120477

**Published:** 2012-10

**Authors:** Jane Kuypers, Angela P. Campbell, Katherine A. Guthrie, Nancy L. Wright, Janet A. Englund, Lawrence Corey, Michael Boeckh

**Affiliations:** University of Washington, Seattle, Washington, USA (J. Kuypers, A.P. Campbell, N.L. Wright, J.A. Englund, L. Corey, M. Boeckh);; Fred Hutchinson Cancer Research Center, Seattle (J. Kuypers, A.P. Campbell, K.A. Guthrie, J.A. Englund, L. Corey, M. Boeckh);; and Seattle Children’s Hospital, Seattle (A.P. Campbell, J.A. Englund)

**Keywords:** human polyomaviruses, viruses, WU polyomavirus, WUPyV, KI polyomavirus, KIPyV, hematopoietic cell transplant, immunocompromised patients, respiratory viruses, respiratory samples

## Abstract

Data are limited regarding 2 new human polyomaviruses, KI polyomavirus (KIPyV) and WU polyomavirus (WUPyV), in immunocompromised patients. We used real-time PCR to test for these and 12 respiratory viruses in 2,732 nasal wash samples collected during the first year after allogeneic hematopoietic cell transplantation from 222 patients. Specimens were collected weekly until day 100; then at least every 3 months. One year after hematopoietic cell transplantation, the cumulative incidence estimate was 26% for KIPyV and 8% for WUPyV. Age <20 years predicted detection of KIPyV (hazard ratio [HR] 4.6) and WUPyV (HR 4.4), and detection of a respiratory virus in the previous 2 weeks predicted KIPyV detection (HR 3.4). Sputum production and wheezing were associated with detection of KIPyV in the past week and WUPyV in the past month. There were no associations with polyomavirus detection and acute graft versus host disease, cytomegalovirus reactivation, neutropenia, lymphopenia, hospitalization, or death.

## Abstract 2

Routine testing for these viruses in immunocompromised patients is not recommended.

In 2007, two new human polyomaviruses, KI polyomavirus (KIPyV) and WU polyomavirus (WUPyV), were identified in respiratory specimens from patients with respiratory illness (*1*,*2*). Since then, KIPyV and WUPyV have been frequently detected in respiratory specimens, especially those from children with respiratory symptoms and from patients co-infected with a respiratory virus (*3*–*11*). However, KIPyV and WUPyV were detected at similar rates in specimens from symptomatic patients and in persons without respiratory symptoms (*6*,*8*,*12*,*13*), suggesting that these viruses might not cause respiratory illness in immunocompetent children.

Two other human polyomaviruses, BK and JC, cause mild or asymptomatic primary infections early in life, followed by persistent, subclinical infections in healthy persons (*14*–*16*). However, these viruses can reactivate, primarily from the kidney, bone marrow, and lymphoid tissue, and cause serious disease in immunocompromised patients (*14*–*16*). Similarly, reactivation of KIPyV and WUPyV from lymphoid tissue was described among immunosuppressed persons with AIDS, although clinical consequences of reactivation were not examined (*17*).

Because KIPyV and WUPyV are frequently detected in association with respiratory symptoms, inhalation is suspected as a potential route of transmission. If KIPyV and WUPyV are respiratory pathogens, they may be more likely to cause respiratory illness in immunocompromised persons, either during primary infection or reactivation. Few, mostly retrospective, studies on the prevalence of KIPyV and WUPyV in respiratory specimens have included large numbers of immunocompromised patients (*18*–*21*). No prospective data are available that comprehensively describe the incidence, symptoms, risk factors, and outcomes associated with detection of KIPyV and WUPyV in respiratory specimens from hematopoietic cell transplant (HCT) recipients. This question is particularly relevant with the increasing use of multiplex PCR panels for detection of respiratory viruses, especially in samples from immunocompromised patients.

To investigate whether respiratory detection of these new polyomaviruses is associated with specific outcomes in patients after HCT, a real-time PCR specific for KIPyV and WUPyV DNA was developed and used to examine nasal wash specimens collected prospectively from HCT recipients with and without respiratory symptoms for 1 year after transplantation. Clinical data and standardized symptom surveys obtained at each specimen collection were analyzed to determine associations between respiratory KIPyV and WUPyV detection and illness.

## Methods

### Patients and Collection of Specimens

Combined nasopharyngeal wash (or swab) and oropharyngeal swab samples were collected weekly beginning 1–2 weeks before transplantation until day 100; then every 1–3 months for ≤1-year after transplantation from allogeneic HCT recipients enrolled in a prospective surveillance study approved by the Institutional Review Board at Fred Hutchinson Cancer Research Center (Seattle, WA, USA) (*22*). Participants provided written informed consent. Additional specimens were collected when respiratory symptoms were reported. Patients had ≥1 specimen collected during January 2006–December 2007. Participants completed surveys weekly for 1 year and reported any of 11 respiratory or 4 systemic symptoms. Clinical and laboratory data were obtained from medical records.

### Detection of KIPyV and WUPyV DNA and PCR Validation

An in-house, duplex, real-time TaqMan PCR was developed, which was specific for viral protein 2–3 and viral protein 1 genes of KIPyV and WUPyV, respectively. Ten microliters of extracted sample were added to the PCR master mixture containing KIPyV and WUPyV primers and probes (Table 1). Samples with PCR cycle threshold values >40 were considered negative. Viral copies per milliliter were determined by using standard curves generated by PCR amplification of 10-fold dilutions of plasmids containing amplicon sequences ranging in concentration from 10 to 1 ×10^7^ copies/reaction. To validate this PCR, a subset of KIPyV-positive, WUPyV-positive, KIPyV-negative, and WUPyV-negative samples was blindly retested by using published assays for detection of KIPyV (*23*) and WUPyV (*4*).

**Table 1:** Primers and probes used for detecting KIPyV, WUPyV, and 2 other viruses by real-time RT-PCR and PCR in HCT recipients*

Virus	Target gene	Amplicon size, bp	Function	Sequence/label, 5′→3′	PCR concentration, nmol/L
KIPyV	VP2–3	74	Forward primer	CTATCCCTGAATACCAGTTGGAAAC	425
			Reverse primer	GTATGACGCGACAAGGTTGAAG	425
			Probe	FAM-TTCCGGGCATCCCAGACTGGC-BHQ1	125
WUPyV	VP1	75	Forward primer	AACCAGGAAGGTCACCAAGAAG	300
			Reverse primer	TCTACCCCTCCTTTTCTGACTTGT	300
			Probe	HEX-CAACCCACAAGAGTGCAAAGCCTTCC-BHQ1	75
Influenza B	Matrix	76	Forward primer	CACAATTGCCTACCTGCTTTCA	250
			Reverse primer	CCAACAGTGTAATTTTTCTGCTAGTTCT	250
			Probe	VIC-CTTTGCCTTCTCCATCTT-MGBNFQ	100
Parainfluenza type 4	NP	94	Forward primer	TGCCAAATCGGCAATAAACA	250
			Reverse primer	GGCTCTGGCAGCAATCATAAG	250
			Probe	VIC-TGATTCTGCATTGATGTGG-MGBNFQ	100

*KIPyV, KI polyomavirus; WUPyV, WU polyomavirus; RT-PCR, reverse transcription PCR; HCT, hematopoietic cell transplantation; VP, viral protein; NP, nucleoprotein.

The in-house PCR had a sensitivity of 5–10 DNA copies/PCR, which provided a sensitivity of 500–1,000 copies/mL. This PCR did not detect JC or BK virus DNA. A subset of 397 samples, including 31 positive for KIPyV and 27 positive for WUPyV by the PCR, was retested by using published real-time PCRs. Samples with discordant KIPyV results included 3 positive by the in-house PCR and negative by the alternate PCR (*23*) and 1 negative by the in-house PCR and positive by the alternate PCR. Samples with discordant WUPyV results included 4 positive by the in-house PCR and negative by the alternate PCR (*4*) and 1 negative by the in-house PCR and positive by the alternate PCR. Samples with discordant results had <5,000 copies/mL, indicating low levels of DNA near the assay limits of detection.

### Detection of Respiratory Virus

Samples were tested for 12 respiratory viruses by using a multiplexed panel of real-time, TaqMan reverse transcription PCRs. Assays to detect respiratory syncytial virus; human metapneumovirus; influenza virus A; parainfluenza viruses 1, 2, and 3; adenoviruses; coronaviruses; rhinoviruses; and bocavirus were performed as described (*24*–*30*). Assays to detect influenza virus B and parainfluenza virus 4 were performed by using the same reagents and thermocycling conditions as the other respiratory virus assays but by substituting the specific primer and probe sets (Table 1). The sensitivity of each assay was 1,000 viral copies/mL. Throughout this report, respiratory virus refers to any of these 12 viruses.

### Statistical Analysis

The probability of detecting KIPyV or WUPyV DNA in ≥1 nasal wash sample from transplantation to 1-year after transplantation was estimated by using cumulative incidence curves. Patient records were censored at 14 days past the time of last eligible respiratory sample, death, or 1 year after transplantation, whichever occurred first. Death before 1 year and within 14 days after the last sample of a patient was treated as a competing risk for detection.

The association of KIPyV and WUPyV detection with respiratory virus positivity was estimated by using a logistic regression model. Differences in KIPyV and WUPyV copies/mL according to patient age at transplantation and co-occurrence of a respiratory virus were assessed by using linear regression. For both model types, robust standard errors were calculated to account for correlation within repeated measures for the same person.

Cox regression models were fit to evaluate potential risk factors for detection of KIPyV or WUPyV in the first year after transplantation, including age, sex, disease risk (standard or high) (*29*,*31*), stem cell source, donor type, conditioning regimen (myeloablative versus nonmyeloablative), donor and recipient cytomegalovirus (CMV) serostatus, grades 2–4 graft versus host disease (GvHD), and respiratory virus detection. Donor CMV serostatus was defined as negative for cord blood recipients. Acute GvHD and respiratory virus detection were treated as time-dependent covariates; respiratory virus detection was set to 1 when ≥1 respiratory virus had been detected in the previous 2 weeks and 0 otherwise. Cox regression models were fit to evaluate risk factors for KIPyV or WUPyV detection in the first 100 days after transplantation.

CMV reactivation was modeled as a time-dependent indicator defined as any antigenemia or positive PCR value, or antigenemia >10 cells per slide or >100 copies/mL by PCR. Neutropenia was modeled as a time-dependent covariate set to 1 when the absolute neutrophil count was <500 cells/mm^3^. When values were missing, the indicator for the previous day was carried forward. Lymphopenia was modeled similarly with 2 thresholds, 100 and 300 cells/mm^3^.

We evaluated detection of KIPyV or WUPyV as predictors of clinical outcomes, including diagnosis of grades 2–4 acute GvHD, CMV reactivation, neutropenia, lymphopenia, and hospitalization within 100 days; and viral symptoms, increased liver transaminase and total bilirubin levels, and death within 1 year. For time-to-event outcomes, patient records were censored at date of last contact or death, and KIPyV and WUPyV detection were treated as time-dependent covariates in Cox regression models. Potential confounders for these models included age, sex, donor type, stem cell source, and conditioning regimen.

Outcomes with multiple occurrences over time were analyzed as functions of recent KIPyV or WUPyV detection by using logistic and linear regression models. Virus detection within the last week was defined as a positive sample at the current or last study contact and a lag time ≤14 days. Virus detection within the last month was analyzed for wheezing and cough because these symptoms may persist, and was defined as a positive sample at the current or any of the last 4 study contacts, as long as those contacts occurred within 45 days. Models were adjusted for detection of respiratory virus within the last week, day relative to transplantation, age, conditioning regimen, donor type, and acute GvHD. Measurements based on multiple patient contacts were entered as repeated measures and adjusted for possible correlation between values within a person by using generalized estimating equations.

For regression models, p values were obtained by using the Wald test; no adjustments were made for multiple comparisons. Two-sided p values <0.05 were considered significant. Analyses were performed by using SAS version 9.0 (SAS Institute, Cary, NC, USA).

## Results

### Patient Characteristics and Detection of KIPyV and WUPyV

A total of 2,732 nasal wash specimens were collected from 222 eligible patients at weekly or longer intervals (median 7 days, range 4–155 days). The cohort had a median of 13 specimens per patient (range 1–30 specimens). Patients ranged in age from 9.6 months to 75.2 years (median 51.5 years) and remained in the study a median of 111 days (range 1–365 days) after HCT. A respiratory virus was detected in 17% of specimens; 51% of patients had ≥1 sample positive for any respiratory virus during their time in the study. Clinical characteristics of the cohort are provided in Table 2.

**Table 2:** Characteristics of 222 HCT recipients tested for KIPyV or WUPyV*

Characteristic	No. (%) recipients		
	All, n = 222	Infected with either virus, n = 62	Not infected, n = 160
Age, y			
<20	23 (10)	18 (29)	5 (3)
20–39	37 (17)	14 (23)	23 (14)
40–59	99 (45)	17 (27)	82 (51)
>60	63 (28)	13 (21)	50 (31)
Sex			
F	87 (39)	23 (37)	64 (40)
M	135 (61)	39 (63)	96 (60)
Underlying disease risk†			
Standard	142 (64)	40 (65)	102 (64)
High	80 (36)	22 (35)	58 (36)
Stem cell source			
Bone marrow	28 (13)	12 (19)	16 (10)
Peripheral blood	179 (81)	47 (76)	132 (83)
Cord blood	15 (7)	3 (5)	12 (8)
CMV serostatus			
D+/R+	56 (25)	17 (27)	39 (24)
D+/R–	81 (36)	22 (35)	59 (37)
D–/R+	16 (7)	4 (6)	12 (8)
D–/R–	69 (31)	19 (31)	50 (31)
Donor match			
Related–matched	77 (35)	23 (37)	54 (34)
Related–mismatched	9 (4)	6 (10)	3 (2)
Unrelated–cord blood	15 (7)	3 (5)	12 (8)
Unrelated–matched	97 (44)	24 (39)	73 (46)
Unrelated–mismatched	24 (11)	6 (10)	18 (11)
Conditioning regimen			
Myeloablative	128 (58)	41 (66)	87 (54)
Nonmyeloablative	94 (42)	21 (34)	73 (46)
Acute graft-versus-host disease			
Grade 0 or 1	78 (35)	30 (48)	48 (30)
Grade 2–4	144 (65)	32 (52)	112 (70)

*HCT, hematopoietic cell transplantation; KIPyV, KI polyomavirus; WUPyV, WU polyomavirus; CMV, cytomegalovirus; D, donor; R, recipient. †The underlying disease stage of a patient was categorized as low, intermediate, or high risk (*29**,**31*).

**Figure 1:**
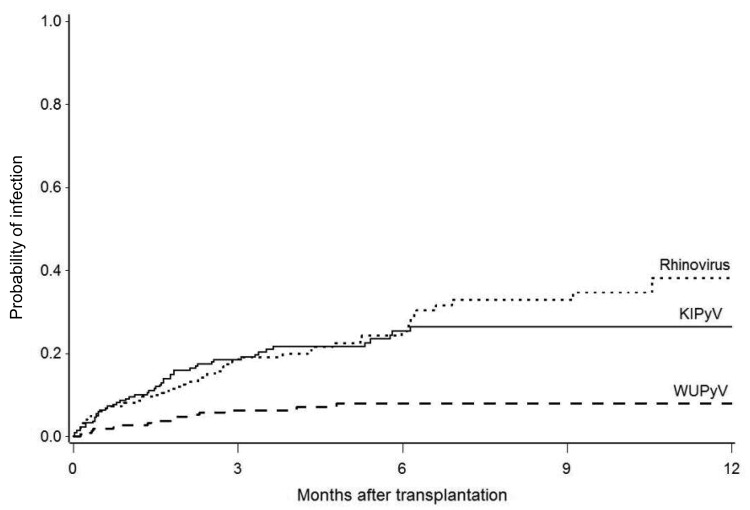
Cumulative incidence of KI polyomavirus (KIPyV) and WU polyomavirus (WUPyV) detection after transplantation in 222 hematopoietic cell transplantation recipients. Cumulative incidence of human rhinovirus is shown for comparison.

**Figure 2:**
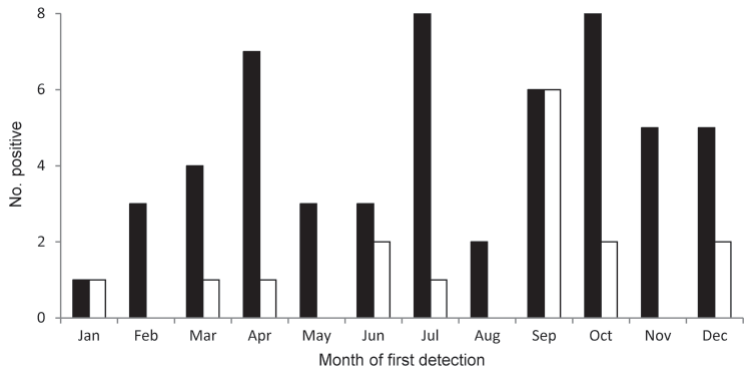
Number of samples positive for KI polyomavirus (black bars) and WU polyomavirus (white bars) by month of first detection in hematopoietic cell transplantation recipients.

KIPyV and WUPyV DNA was detected in 203 (7%) specimens from 49 (22%) patients and in 35 (1%) specimens from 15 (7%) patients, respectively. Two patients were positive for both viruses; 1 patient was positive concurrently in the same specimen, and an additional patient was positive for 1 virus in different specimens. Among the 62 WUPyV-positive or KIPyV-positive patients, 1 patient provided only 1 specimen during the study. At 1 year after HCT, cumulative incidence estimates were 26% (95% CI 20%–33%) for KIPyV and 8% (95% CI 4%–12%) for WUPyV (Figure 1). KIPyV and WUPyV were first detected a median of 45 days (range 1–187 days) and 43 days (range 4−46 days) after transplantation, respectively. KIPyV-positive or WUPyV-positive specimens were detected in every month, and there was no apparent seasonality (Figure 2). A respiratory virus was detected in 32% of KIPyV-positive and 14% of WUPyV-positive specimens. Rhinoviruses and coronaviruses accounted for 78% of respiratory virus co-detections. Respiratory and KIPyV viruses tended to co-occur (odds ratio [OR] 2.4, 95% CI 1.2–5.1, p = 0.02).

**Figure 3:**
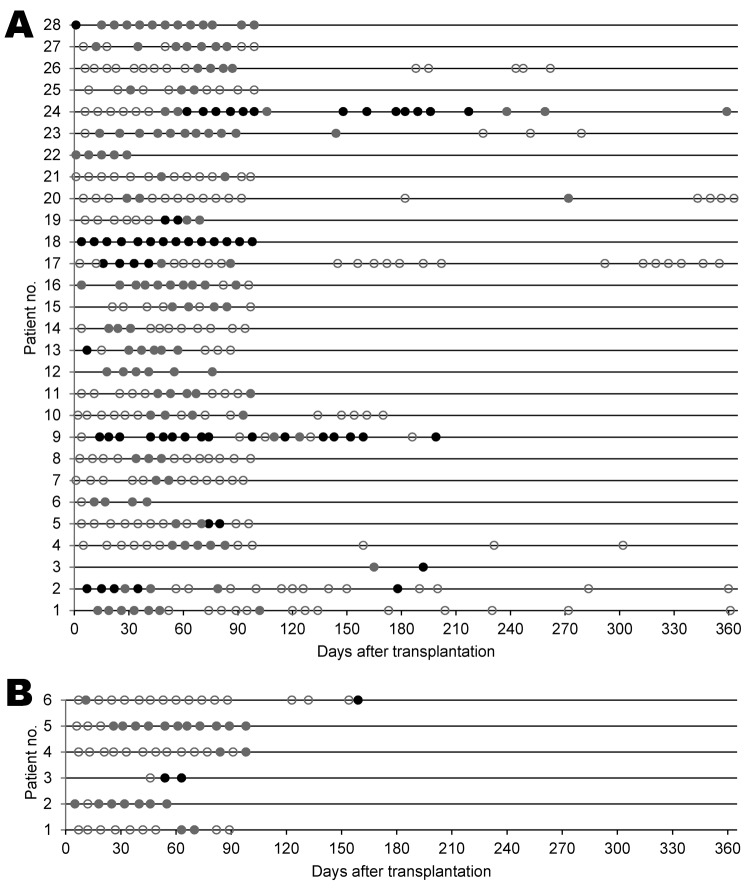
Detection of A) KI polyomavirus (KIPyV) DNA in 28 hematopoietic cell transplantation (HCT) recipients with >2 KIPyV-positive specimens and B) WU polyomavirus (WUPyV) DNA in 6 HCT recipients with >2 WUPyV-positive specimens, with and without detection of a respiratory virus by day after transplantation. Each line represents 1 patient in order of age (KIPyV-positive patients 1–10 and WUPyV-positive patients 1 and 2 are <20 years of age). Circles indicate specimen collection. Gray indicates detection of only KIPyV or WUPyV, black indicates detection of KIPyV or WUPyV and a respiratory virus, and white indicates negative results for KIPyV or WUPyV.

Twenty-one (43%) KIPyV-positive and 9 (60%) WUPyV-positive patients had 1 positive specimen. Positive episodes with detection in ≥4 consecutive specimens were seen in 18 (37%) KIPyV-positive patients, including 6 with 9–19 consistently positive specimens (Figure 3, panel A) and in 2 (13%) WUPyV-positive patients, including 1 with 11 consecutive specimens (Figure 3, panel B). The maximum number (log_10_ copies/mL) of KIPyV and WUPyV virus per positive episode ranged from 2.55 to 10.58 (median 5.31) and 2.57–9.02 (median 3.08), respectively. Patients with >1 KIPyV-positive specimen had an average of >3 logs higher maximum viral log_10_ copies/mL (median 6.56) than KIPyV-positive patients with 1 positive specimen (median 2.86, p<0.001).

If we considered all positive samples, the number of KIPyV copies/mL was significantly higher in specimens positive for KIPyV and a respiratory virus than in specimens in which KIPyV was the only virus detected (medians 6.35 vs. 4.25, respectively, p<0.001). In contrast, WUPyV-positive specimens with a respiratory virus co-pathogen had significantly lower viral copy numbers than specimens positive for WUPyV virus alone (medians 2.84 vs. 3.62, respectively, p = 0.01). Patient age at transplantation was not correlated with KIPyV or WUPyV copies/mL.

### Risk Factors for Detection of KIPyV and WUPyV

**Figure 4:**
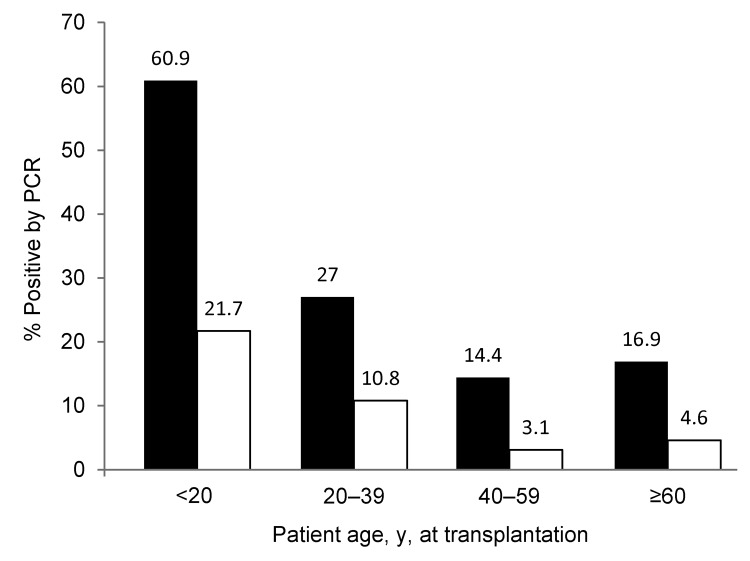
Proportion of hematopoietic cell transplantation recipients in each age group with samples positive for KI polyomavirus (black bars) and WU polyomavirus (white bars). Values above bars are percentages.

Time in study and number of specimens collected did not differ among patients according to polyomavirus detection. Age <20 years was a significant predictor of KIPyV and WUPyV detection in multivariable models (hazard ratio [HR] 4.6, 95% CI 2.5–8.6, p<0.001; HR 4.4, 95% CI 1.5–12.8, p = 0.007; respectively). All 10 patients <12 years of age and 18 (78%) of 23 patients <20 years of age were positive for KIPyV or WUPyV compared with 44 (22%) of 199 patients ≥20 years of age (Figure 4). Detection of a respiratory virus within the last 2 weeks was a significant predictor of KIPyV detection (HR 3.4, 95% CI 1.8–6.4, p<0.001) in a model adjusted for age, transplantation type, and donor type. CMV reactivation, neutropenia, and lymphopenia were not associated with detection of KIPyV and WUPyV within the first 100 days after transplantation.

### Associations between KIPyV or WUPyV Detection and Symptoms

Detection of KIPyV within the past week was significantly associated with sputum production (OR 1.7, 95% CI 1.0–2.9, p = 0.04) (Table 3). WUPyV detection within the past month was significantly associated with wheezing (OR 3.1, 95% CI 1.2–8.1, p = 0.02). Limiting the analysis to 20 patients with high levels of KIPyV detection within the past week (>5 log_10_ copies/mL) showed a significant association with sputum production (OR 2.0, 95% CI 1.1–3.8, p = 0.03). Analysis of symptoms as a function of KIPyV positivity among 17 patients with a respiratory virus detected within the past week showed a similar association between sputum production and KIPyV detection (OR 2.7, 95% CI 1.1–6.6, p = 0.03).

**Table 3:** Respiratory and systemic symptoms associated with KIPyV or WUPyV detection within the prior week or month in HCT recipients*

Symptom	KIPyV†			WUPyV†	
	OR (95% CI)	p value		OR (95% CI)	p value
Within prior week					
Runny nose	1.3 (0.7–2.4)	0.39		1.2 (0.5–3.2)	0.66
Sinus congestion	0.8 (0.5–1.3)	0.34		1.4 (0.6–3.5)	0.46
Postnasal drip	0.8 (0.4–1.8)	0.65		1.2 (0.5–3.0)	0.72
Shortness of breath	0.9 (0.4–1.8)	0.73		0.9 (0.3–2.9)	0.83
Sputum production	1.7 (1.0–2.9)	0.04		1.5 (0.5–4.8)	0.47
Pharyngitis	1.0 (0.6–1.7)	0.99		2.1 (0.8–5.4)	0.12
Sneezing	1.2 (0.7–2.0)	0.54		1.3 (0.6–2.9)	0.48
Watery eyes	1.5 (0.8–2.6)	0.17		1.6 (0.4–6.2)	0.51
Cough	1.1 (0.6–2.0)	0.68		1.4 (0.6–3.5)	0.47
Wheezing	1.1 (0.5–2.5)	0.82		2.1 (0.8–5.9)	0.15
Fever	0.9 (0.5–1.5)	0.59		1.3 (0.4–3.7)	0.64
Headache	0.6 (0.4–1.1)	0.08		0.7 (0.2–2.8)	0.58
Myalgia	0.6 (0.3–1.1)	0.08		1.1 (0.3–3.5)	0.92
Diarrhea	0.8 (0.5–1.3)	0.40		0.7 (0.3–1.8)	0.47
Within prior month					
Cough	1.0 (0.6–1.8)	0.91		1.6 (0.7–3.5)	0.26
Wheezing	1.0 (0.5–2.2)	0.99		3.1 (1.2–8.1)	0.02

*Values are for 211 of 222 patients with symptom survey data. Ear pain was too rare for analysis. KIPyV, KI polyomavirus; WUPyV, WU polyomavirus; HCT, hematopoietic cell transplantation; OR, odds ratio. †Each result was adjusted for the following covariates: detection of the other polyomavirus (KIPyV or WUPyV), detection of a respiratory virus, day relative to transplantation, age at transplantation, stem cell source, donor type, and grades 2–4 acute graft-versus-host-disease.

### Associations between KIPyV or WUPyV Detection and Clinical Outcomes

Longitudinal analyses showed significant relationships between WUPyV detection and a lower risk for lymphopenia defined by <300 cells/mm^3^ (OR 0.3, 95% CI 0.1–0.6, p = 0.001) and grades 2–4 acute GvHD (HR 3.1, 95% CI 1.3–7.7, p = 0.01) in a model adjusted for donor type and stem cell source among 136, 27, and 6 patients given diagnoses of grades 2, 3, and 4 acute GvHD, respectively. However, this association was based on few cases; only 5 patients had WUPyV detected before the GvHD diagnosis. No relationships between detection of KIPyV or WUPyV and CMV reactivation, risk for hospitalization ≤100 days after transplantation, and mean values for alanine aminotransferase, aspartate aminotransferase, and total bilirubin levels were found in multivariable models.

Nineteen bronchoalveolar lavage (BAL) samples from 13 KIPyV-positive patients and 57 BAL samples from 37 KIPyV-negative and WUPyV-negative patients were tested for KIPyV and WUPyV. Collection of BAL samples occurred within 2 weeks of a positive nasal wash sample in only 4 of the KIPyV-positive patients, including 3 with collection on the same day. Only 1 of 76 BAL samples was positive for KIPyV (6.3 log_10_ copies/mL). This sample was collected from a 2-year-old child on the same day as the KIPyV-positive nasal wash sample (165 days after transplantation); CMV was also isolated in culture and the patient received treatment for CMV pneumonia. Of 66 deaths within 1 year of transplantation, 12 occurred in patients positive for KIPyV, 4 in patients positive for WUPyV, and 1 in a patient positive for KIPyV and WUPyV. Neither virus was associated with the 1-year mortality rate in in an adjusted model.

## Discussion

Our large longitudinal surveillance study provides a rigorous evaluation of KIPyV and WUPyV detection in upper respiratory tract specimens from HCT recipients. One-year cumulative incidence estimates were high, 26% and 8% for KIPyV and WUPyV, respectively, and there were prolonged episodes of detection for ≥4 weeks in 37% of patients positive for KIPyV and 13% of patients positive for WUPyV. These numbers are comparable to those of our report of rhinoviruses and coronaviruses, the most common respiratory virus types detected in the same patient population, in which we found day 100 estimates of 22% and 11%, respectively, and detection for ≥1 month in 44% and 37%, respectively (*29*). In comparison, cross-sectional studies testing specimens from immunocompetent children with acute respiratory tract illnesses found the prevalence of KIPyV and WUPyV ranged from 0% to 2.8% and from 2% to 7.1% (*1*–*13*,*21*).

A recent study of pediatric hematology/oncology patients and immunocompetent persons found a higher KIPyV mean viral load in respiratory tract specimens from the immunocompromised group, suggesting potential for increased pathogenicity in this population (*21*). A study in adult HCT recipients tested sequential nasopharyngeal aspirates from 31 asymptomatic patients, in which KIPyV and WUPyV were detected in 1 each of 126 samples (*19*). A cross-sectional study that tested specimens from 45 HCT recipients with respiratory illness detected KIPyV in 8 (17.8%), similar to our findings, but with no WUPyV detected (*19*,*20*). Respiratory viruses were often co-detected in our cohort, 32% with KIPyV and 14% with WUPyV, consistent with high rates of co-infection reported by others (*1*–*3*,*6*,*10*,*21*,*32*).

In our study, young age was a risk factor for detection of KIPyV or WUPyV, which has been reported (*4*,*7*,*32*,*33*). The seroprevalence of antibodies to KIPyV and WUPyV for children 5–20 years of age was similar to that for adults (*34*,*35*), suggesting that primary exposure occurs in childhood. We also found that detection of a respiratory virus within the previous 2 weeks was an independent risk factor for detection of KIPyV. Compared with samples in which only KIPyV DNA was detected, higher viral copy numbers of KIPyV were detected in samples also positive for a respiratory virus, perhaps because of stimulation of viral replication during a respiratory virus infection. Our current data do not provide evidence that this is a biologically meaningful difference.

Prolonged detection and younger age associated with a higher prevalence of KIPyV and WUPyV were also reported in a longitudinal study of KIPyV and WUPyV in children (*33*). As with BK and JC polyomaviruses, KIPyV and WUPyV might persist for life after primary infection in undetermined sites and become reactivated during periods of immune suppression. Prolonged detection episodes may represent long-term viral shedding after new acquisition of virus, especially in young patients, or reactivation or stimulation of replication of persistent virus in the respiratory tract because of immune suppression or a recent or concomitant respiratory virus infection. A site of persistence for these new polyomaviruses has not yet been identified, although human tonsils have been postulated as a reservoir and reactivation has been demonstrated in lymph nodes and spleen (*17*,*36*,*37*).

The longitudinal design of our study and specimen collection during symptomatic and asymptomatic periods enabled us to thoroughly evaluate symptoms associated with KIPyV and WUPyV detection in HCT recipients. We found that KIPyV detection in the past week was associated with sputum production and WUPyV detection in the past month was associated with wheezing. Limiting the analysis to patients with high viral copy numbers of KIPyV in their nasal wash sample did not provide additional associations. Likewise, co-detection of KIPyV and a respiratory virus was not associated with symptoms other than sputum production, suggesting that infection with KIPyV did not exacerbate symptoms caused by the respiratory virus. Of 50 patients who underwent bronchoscopy for workup of possible lower respiratory illness and had a BAL specimen available for testing, we detected KIPyV in 1 sample from a child who also had CMV pneumonia. Overall, our analyses provide some support for these viruses as respiratory pathogens. However, we did not observe severe respiratory illness or death that could be conclusively attributed to polyomavirus detection.

In seeking other biologically plausible and clinically relevant associations for these new viruses, we found no association of KIPyV and WUPyV detection in nasal washes with CMV reactivation, increased liver enzyme levels, hospitalization, or neutropenia in the first 100 days after transplantation. Detection of WUPyV was associated with a lower risk for lymphopenia.

We did not test other samples, such as blood, urine, or feces, for KIPyV and WUPyV DNA. Previous studies reported detection of these viruses in plasma, serum, and peripheral blood samples from patients infected with HIV-1, healthy blood donors, and children and in urine samples from children (*1*,*2*,*38*) and immunocompetent and immunocompromised adults (*18*,*38*). WUPyV was detected in serum and feces of children whose nasopharyngeal aspirate samples contained high viral loads (*39*). Both viruses were detected in fecal samples from HCT recipients (*18*,*40*), and an association was found between KIPyV and diarrhea (*40*). However, we did not find an association between virus and diarrhea. Another limitation is that although we report prolonged, uninterrupted KIPyV and WUPyV detection, we did not perform genetic analyses to confirm whether these detections represent the same or different viral subtypes.

In conclusion, we detected KIPyV or WUPyV in one third of allogeneic HCT recipients during the first year after transplantation. Prolonged detection episodes and high viral copy numbers in respiratory specimens were also observed. However, we did not observe many associations with acute respiratory symptoms. We found that detection of a respiratory virus was a risk factor for KIPyV detection and that concurrent detection of the polyomaviruses and respiratory viruses was common. Although it has been suggested that KIPyV and WUPyV might be major pathogens in immunosuppressed patients, we did not find a clear role for these viruses as respiratory pathogens with classic upper and lower respiratory tract symptoms. At this time, we do not recommend routine testing for these viruses in immunocompromised patients or inclusion in multiplexed respiratory virus PCR panels. Further investigations examining other specimen types, such as BAL, blood, and urine, from a larger patient cohort over a longer period of time may be necessary to elucidate the role of these viruses in highly immunocompromised patients.
